# Characterization of INS-15, A Metalloprotease Potentially Involved in the Invasion of *Cryptosporidium parvum*

**DOI:** 10.3390/microorganisms7100452

**Published:** 2019-10-14

**Authors:** Rui Xu, Yaqiong Guo, Na Li, Qiang Zhang, Haizhen Wu, Una Ryan, Yaoyu Feng, Lihua Xiao

**Affiliations:** 1 State Key Laboratory of Bioreactor Engineering, School of Resources and Environmental Engineering, East China University of Science and Technology, Shanghai 200237, China; ruix@wustl.edu (R.X.); zhangqiang1991414@gmail.com (Q.Z.); 2 Key Laboratory of Zoonosis of Ministry of Agriculture, College of Veterinary Medicine, South China Agricultural University, Guangzhou 510642, China; guoyq@scau.edu.cn (Y.G.); nli@scau.edu.cn (N.L.); 3 School of Biotechnology, East China University of Science and Technology, Shanghai 200237, China; wuhzh@ecust.edu.cn; 4 College of Science, Health Education and Engineering, Vector- and Water-Borne Pathogen Research Group, Murdoch University, Murdoch, Western Australia 6150, Australia; una.ryan@murdoch.edu.au

**Keywords:** *Cryptosporidium parvum*, insulinase-like protease, expression, invasion

## Abstract

*Cryptosporidium parvum* is a protozoan parasite that can cause moderate-to-severe diarrhea. Insulinase-like proteases (INS) are one of the largest protein families within the small proteome of the pathogen. However, their roles in *C. parvum* biology remain un-elucidated. In this study, a member of the protein family, INS-15 of *C. parvum* encoded by *cgd3_4260*, was cloned, expressed and characterized to understand its function. INS-15 and its domain I were expressed in *Escherichia coli* and polyclonal antibodies against the domain I and one specific polypeptide were prepared in rabbits. The role of INS-15 protein in the *C. parvum* invasion was preliminarily studied. Recombinant INS-15 protein and its domain I were successfully expressed in *E. coli*, together with various degraded products. The *cgd3_4260* gene had a peak expression at 2 h of *in vitro* *C. parvum* culture, while the INS-15 protein was expressed in the mid-anterior region of sporozoites and the area of merozoites opposite to the nucleus. Anti-INS-15 domain I antibodies reduced the invasion of *C. parvum* sporozoites by over 40%. The anterior location of INS-15 in invasion stages and partial reduction of *in vitro* growth indicate that INS-15 plays some roles in the invasion or early development of *C. parvum*.

## 1. Introduction

*Cryptosporidium* spp. are apicomplexan parasites that have emerged as an important cause of diarrhea in humans and animals [1]. They infect all vertebrates, causing watery diarrhea in young animals, especially pre-weaned calves and lambs [2]. In addition, cryptosporidiosis is one of the top five causes of moderate-to-severe diarrhea in children younger than two years in developing countries [3,4]. In 2016 alone, acute cryptosporidiosis in children under five has led to more than 4.2 million disability-adjusted life-years (DALYs) lost and more than 48,000 deaths globally [5]. Most human *Cryptosporidium* infections are caused by *C. hominis* and *C. parvum*, which differ in host range [6]. The former mainly infects humans while the latter infects humans, ruminants and some other animals.

The lack of effective drugs against cryptosporidiosis is largely due to our limited understanding of the biology of *Cryptosporidium* spp., especially the invasion process. Nitazoxanide is the only drug approved by the US Food and Drug Administration (FDA) for the treatment of cryptosporidiosis, but it is ineffective in immunocompromised individuals [7]. *Cryptosporidium* spp. have a complex life cycle including excystation, adhesion, invasion, and intracellular multiplication during early stages of the infection [8]. In addition, the molecular mechanisms of the invasion in *Cryptosporidium* spp. appear to be significantly different from *Toxoplasma gondii* and *Plasmodium* spp. [9]. To facilitate the development of effective control measures, efforts have been made to identify and characterize proteins involved in the early interactions between the pathogens and host cells, with over 30 candidates being identified thus far [10,11].

Secreted proteases and protein kinases released by secretory organelles of apicomplexans are known to be involved in processing invasion-related proteins or modifying host cell activities during the invasion of the host [12]. Insulinase-like proteases (INS) belonging to the M16 superfamily of metalloproteases are members of this large group of enzymes. A classic M16 protease normally contains four domains: one active domain characterized by the presence of an inverted zinc-binding motif HxxEH (where x can be any amino acid) as well as three inactive catalytic domains [13]. Human insulinase can cleave a variety of peptides, such as insulin, β-endorphin, and amyloid β-protein. INS members are reportedly localized in cytosol, peroxisomes, endosomes and even on the surface of cells [14], suggesting a broad range of functions by these enzymes. INS proteins have also been found in other apicomplexans, such as *T. gondii* [15,16] and *Plasmodium falciparum* [17]. They may play important roles in the invasion and early development of these pathogens.

Results of comparative genomics analyses indicate that INS proteases are common in *Cryptosporidium* spp., with 12–22 genes being identified in different species, including INS-15 and INS-16. For example, *C. parvum* has 22 INS proteins [10], 13 of which are expressed at high levels during early infection [18]. Most of them belong to the M16A subfamily, except for two M16B proteins and one M16C protein. Although *C. hominis* is genetically related to *C. parvum*, at least two of the INS genes are missing in its genome, suggesting that INS could play a potential role in determining some phenotypic differences between the two species [10]. A recent study of one *C. parvum*-specific INS, the INS-20-19 encoded by a subtelomeric gene (*cgd6_5520_5510*) in chromosome 6, has shown that indeed these proteins could be involved in the invasion and early growth of the pathogen [19]. INS-20-19, however, does not have all 4 domains present in classic INS.

In this study, we have focused on the INS-15 encoded by the *cgd3_4260* gene. It was chosen because it is encoded by one member of the 11-gene cluster on chromosome 3 and has all 4 domains that are present in classic M16A metalloproteases, other INS proteases in *Cryptosporidium* spp. mostly have one or more domains missing. As INS-15 has significant sequence homology (90% nucleotide sequence identity) to INS-16 encoded by the *cgd3_4270* gene, which is absent in *C. ubiquitum*, it may have important biologic functions in *Cryptosporidium* spp.

## 2. Results

### 2.1. Expression of Recombinant INS-15 and INS-15 Domain I

**Figure 1:**
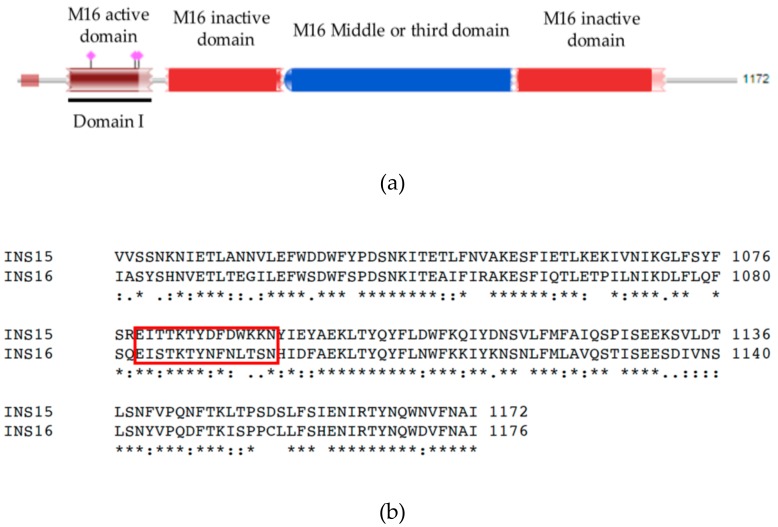
Sequence features of INS-15. (**a**) Diagram of INS-15 of *Cryptosporidium parvum* illustrating the presence of one M16 active domain, two inactive domains, and one middle or third domain. The black line identifies the domain I. (**b**) Alignment of partial amino acid sequences of INS-15 and INS-16 from *C. parvum*. The red box shows the differences in amino acid sequences between NS-15 and INS-16 in the area of INS-15-specific polypeptide used in the study.

**Figure 2:**
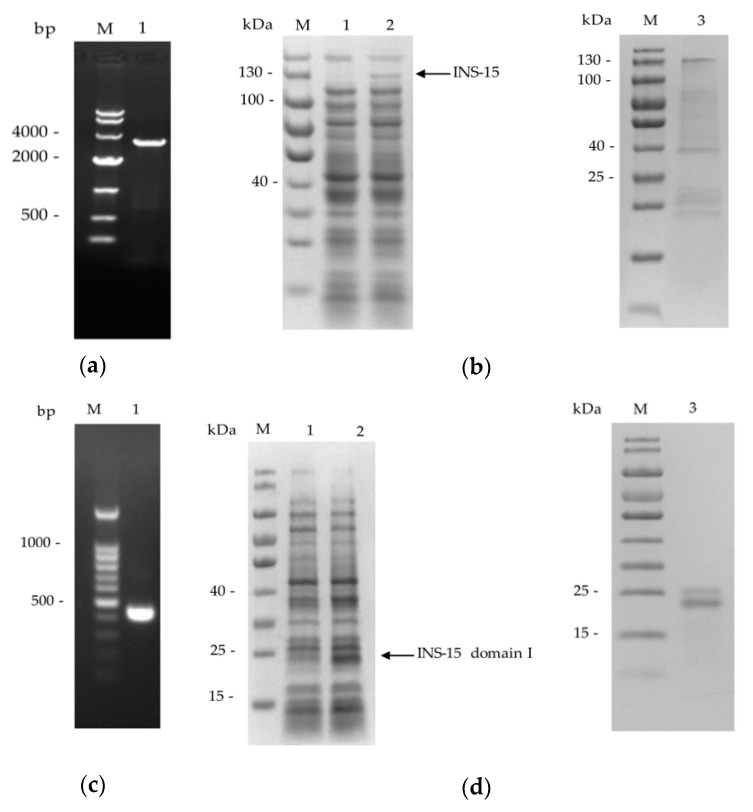
Expression of full-length INS-15 and its domain I in *Escherichia coli*. (**a**) PCR amplification of the *cgd3_4260* gene of *C. parvum*. Lane M: 1000 bp molecular makers, Lane 1: *cgd3_4260* product. (**b**) SDS-PAGE analysis of full-length INS-15 expressed in *E. coli* (left panel) and purified INS-15 protein from *E. coli* (right panel). Lane M: molecular weight markers, Lane 1: lysate from recombinant bacteria without isopropyl β-D-thiogalactoside (IPTG) induction, Lane 2: lysate from recombinant bacteria after IPTG induction, with the expected product indicated by an arrow, Lane 3: INS-15 purified from the *E. coli* lysate using Ni-NTA affinity chromatography. (**c**) PCR amplification of the domain I fragment of the *cgd3_4260* gene of *C. parvum*. Lane M: 100 bp molecular markers, Lane 1: *cgd3_4260* domain I product. (**d**) SDS-PAGE analysis of recombinant INS-15 domain I expressed in *E. coli* (left panel) and purified INS-15 domain I protein from *E. coli* (right panel). Lane M: molecular weight markers, Lane 1: lysate from *E. coli* culture without IPTG induction, Lane 2: lysate from *E. coli* culture after IPTG induction, with the expected product being indicated by an arrow, Lane 3: INS-15 domain I purified from the lysate using Ni-NTA affinity chromatography.

The full *cgd3_4260* gene and its domain I fragment (Figure 1a) were amplified by PCR (Figure 2a,c) and cloned into the pET-28a vector. The recombinant INS-15 and INS-15 domain I generated were purified using the His-tag incorporated (Figure 2b,d). In SDS-PAGE analysis of the recombinant INS-15, the expected band with the predicted size of ~130 kDa was seen. However, there were also several bands smaller than 130 kDa. The INS-15 identity of the 130 kDa, ~40 kDa, ~25 kDa bands were confirmed using MALDI-TOF-MS analysis, yielding peptide sequences of INS-15 (Accession No. XM_626969, date not shown). In SDS-PAGE analysis of the INS-15 domain I, the expected band of the predicted size of ~25 kDa was seen. Similarly, there was another band of ~23 kDa. The INS-15 identity of both bands was also confirmed by the results of the MALDI-TOF-MS analysis.

### 2.2. Identification of Native INS-15

**Figure 3:**
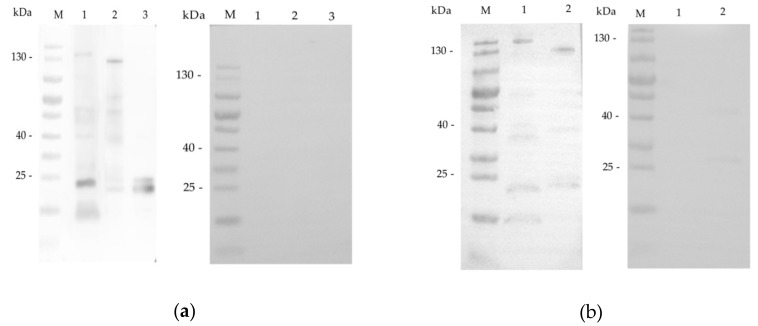
Expression of native INS-15 protein in *C. parvum* sporozoites. (**a**) Western blot analysis of native INS-15 protein in crude sporozoite extract in the presence of protease inhibitors using antibodies against the INS-15 domain I (left panel) and pre-immune serum (right panel). Lane M: molecular weight markers, Lane 1: native proteins from sporozoites, Lane 2: purified full-length recombinant INS-15 protein, Lane 3: purified INS-15 domain I. (**b**) Western blots analysis of native INS-15 protein using anti-INS-15 polypeptide antibodies (left panel) and pre-immune serum (right panel). Lane M: molecular weight markers, Lane 1: native proteins from sporozoites, Lane 2: purified full-length recombinant INS-15 protein.

To characterize native INS-15, antibodies against the INS-15 domain I and an INS-15 polypeptide were generated. In western blot analysis, antibodies against the INS-15 domain I reacted with a protein of ~140 kDa weakly and several proteins smaller than 100 kDa in lysates of *C. parvum* sporozoites, especially a band of ~23 kDa. As expected, they recognized the full-length 130 kDa recombinant protein, fragments of it and two bands of ~25 kDa and ~23 kDa in recombinant INS-15 domain I (Figure 3a). The pre-immune serum did not react with any of these proteins, confirming the specificity of the antibodies to INS-15. Antibodies against INS-15 polypeptide produced similar profiles of reactivity to native and recombinant INS-15 proteins, although the reactivity to the ~23 kDa band was much weaker compared with antibodies against domain I (Figure 3b).

### 2.3. Expression of the INS-15 Gene and Protein in Life Cycle Stages

**Figure 4:**
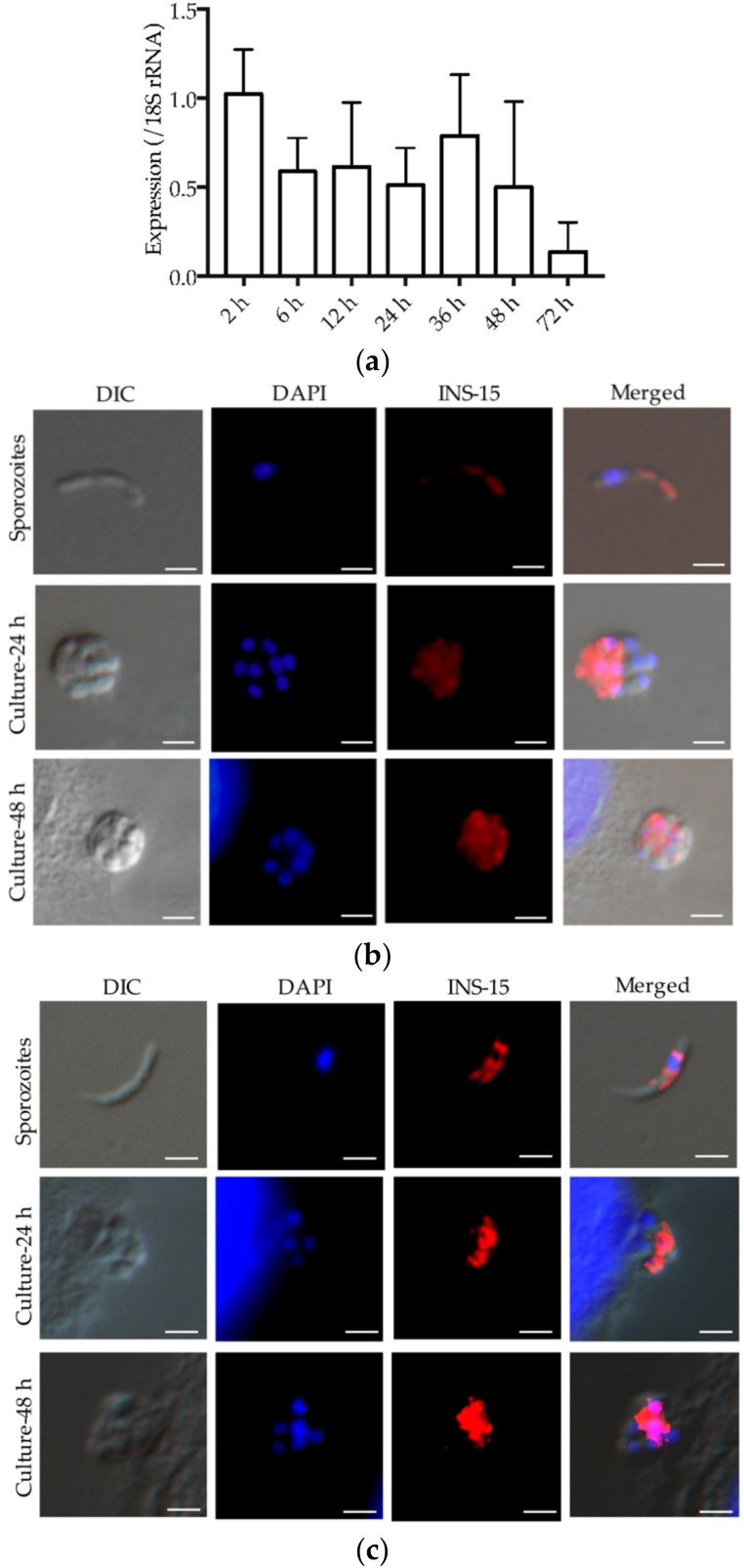
Expression of the INS-15 gene and protein in *C. parvum*. (**a**) Relative expression level of the *cgd3_4260* gene at various *C. parvum* cultivation time as determined by reverse transcription-qPCR. Data from the *Cryptosporidium* 18S rRNA gene were used in data normalization. Data presented are mean ± SD from three replicate assays, with qPCR analysis of each RNA extraction being performed in duplicate. (**b**,**c**) Expression of INS-15 protein in *C. parvum* life cycle stages indicated by immunofluorescence microscopy with anti-INS-15 domain I antibodies (**b**) or anti-INS-15-specific polypeptide antibodies **c**). Sporozoites of *C. parvum* (top panel) used were obtained from excysted oocysts, while intracellular developmental stages were obtained by cultivating *C. parvum* in HCT-8 cells for 24 h (middle panel) and 48 h (bottom panel). The images were taken under differential interference contrast (DIC), with nuclei being counter-stained with 4′, 6-diamidino-2-phenylindole (DAPI), parasites stained by immunofluorescence with anti-INS-15 antibodies, and superimposition of the three (Merged). Scale-bars: 2 μm.

An analysis of the expression of the *cgd3_4260* gene over a 72-h time course of *C. parvum* infection in HCT-8 cells indicated that the highest INS-15 gene expression was at 2 h of the infection (Figure 4a).

The antibodies produced against the INS-15 domain I and polypeptide were used to examine INS-15 expression in *C. parvum* sporozoites and intracellular stages using immunofluorescence microscopy. In sporozoites, anti-INS-15 domain I antibodies reacted with the mid-anterior region (Figure 4b, top panel). At 24 h and 48 h of cell culture, antibodies had the highest reactivity to the part of merozoites opposite to the nucleus (Figure 4b, middle and bottom panel). A similar staining pattern of sporozoites and merozoites were obtained with antibodies to the INS-15-specific polypeptide, although the reactivity was slightly reduced compared with antibodies against domain I (Figure 4c).

### 2.4. Inhibition of C. parvum Invasion by Anti-INS-15 Domain I Antibodies and Anti-INS-15 Polypeptide Antibodies

**Figure 5:**
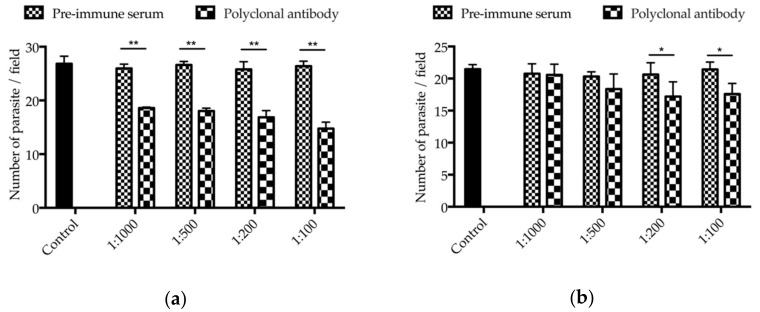
Partial neutralization of *C. parvum* invasion by antibodies against INS-15. Hypochlorite-treated oocysts were pre-incubated with 1:1000, 1:500, 1:200, and 1:100 dilutions of antibodies against the INS-15 domain I (**a**), the INS-15-specific polypeptide antibodies (**b**), with pre-immune serum or culture medium alone as controls. Parasite loads per 200× microscope field were calculated and compared among treatment groups. Data presented are mean ± SD from three replicate assays. The * or ** symbol above the treatment group indicates the difference between the treated and untreated groups is statistically significant at *p* < 0.05 and *p* < 0.01, respectively.

The neutralizing effect of antibodies against INS-15 domain I and polypeptides on *C. parvum* invasion were assessed using *in vitro* cell culture. In comparison with the control culture, the mean parasite load was reduced significantly when the cell culture was inoculated with sporozoites treated with antibodies against INS-15 domain I (Figure 5a). The inhibitory effect was 28.4% (26.0 ± 0.8 and 18.6 ± 0.2 parasites per 200× field for pre-immune serum and antibodies, respectively, *t*_(2)_ = 13.373, *p* = 0.006) at 1:1000 dilution, 32.2% (26.6 ± 0.6 and 18.0 ± 0.5 parasites per 200× field for pre-immune serum and antibodies, respectively, *t*_(2)_ = 107.250, *p* < 0.001) at 1:500 dilution, 34.6% (25.8 ± 1.4 and 16.9 ± 1.2 parasites per 200× field for pre-immune serum and antibodies, respectively, *t*_(2)_ = 14.820, *p* = 0.005) at 1:200 dilution, and 44.0% (26.4 ± 0.9 and 14.8 ± 1.2 parasites per 200× field for pre-immune serum and antibodies, respectively, *t*_(2)_ = 74.389, *p* < 0.001) at 1:100 dilution. In contrast, the inhibitory effect of antibodies to the INS-15 polypeptide was 0.9% (20.8 ± 1.5 and 20.6 ± 1.7 parasites per 200× field for pre-immune serum and antibodies, respectively, *t*_(2)_ = 1.104, *p* = 0.385) at 1:1000 dilution, 9.6% (20.3 ± 0.7 and 18.4 ± 2.4 parasites per 200× field for pre-immune serum and antibodies, respectively, *t*_(2)_ = 1.880, *p* = 0.201) at 1:500 dilution, 16.6% (20.6 ± 1.8 and 17.2 ± 2.3 parasites per 200× field for pre-immune serum and antibodies, respectively, *t*_(2)_ = 8.883, *p* = 0.012) at 1:200 dilution, and 17.9% (21.4 ± 1.1 and 17.6 ± 1.7 parasites per 200× field for pre-immune serum and antibodies, respectively, *t*_(2)_ = 6.352, *p* = 0.024) at 1:100 dilution (Figure 5b).

## 3. Discussion

Insulinase-like proteases have been reported in numerous apicomplexan parasites and appear to play important roles in the invasion and growth of parasites. Among them, falcilysin was found mostly in the apicoplast of *P. falciparum* [20,21], toxolysin-1 was localized in the rhoptry and secreted during host cell invasion of *T. gondii* [15], while toxolysin-4 was localized in the micronemes and secreted in the response to elevated calcium [16]. *Cryptosporidium* spp. appear to have many more INS than other apicomplexans [10]. Thus far, the function of this large family of proteins remains unclear. Recently, we characterized the function of a *C. parvum*-specific INS, INS-20-19, which has only two of the four domains of functional insulinases [19]. In this study, we identified, expressed, and characterized INS-15 of *C. parvum*, which has all four domains.

Data from the present study suggest that INS-15 may participate in the invasion of host cells by *C. parvum*. The INS-15 gene has peak expression during the early period of the life cycle of *C. parvum*. Furthermore, the INS-15 protein is expressed in the mid-anterior region of sporozoites and a part of merozoites opposite to the nucleus, suggesting that INS-15 may play similar roles in invasive stages of the parasite. In agreement with the potential involvement of INS-15 in the invasion of host cells, antibodies against the INS-15 domain I reduced the invasion of HCT-8 cells by over 40%. The neutralization effect of the polyclonal antibodies could be a combined effect on both INS-15 and INS-16, which have high sequence similarity. Nevertheless, the modest reduction in invasion is expected, because *C. parvum* has several other INS with all four domains of functional insulinases, and it is known that most apicomplexans use multiple strategies for invasion [22]. Taken together, these data suggest that INS-15 could be involved in the invasion on the early growth of parasites [23]. Further studies of the subcellular localization of INS-15 and gene ablation using the CRISPR/Cas9 system or gene overexpression are needed to elucidate the exact biological functions of the protein.

In comparison with INS-20-19, INS-15 is a classic insulinase that is present in all *Cryptosporidium* species and has shown a different expression profile. The gene for it is located in a cluster of 11 INS genes in chromosome 3 [10,19]. In contrast, INS-20-19 is a unique protein that is absent in *C. hominis* and has only two of the four domains of functional insulinases. The gene encoding it is located in the subtelomeric region of chromosome 6 [10,19]. Although the INS-20-19 and INS-15 genes both have peak expression at 2 h post-infection *in vitro*, the expression of the INS-20-19 protein appears in the apical region of sporozoites and the entire surface of merozoites, while the INS-15 protein is expressed in the mid-anterior region of sporozoites and possibly also merozoites. In this study, two antibodies were used to localize the expression of INS-15. INS-15 expression was localized to the mid-anterior region of sporozoites and the area of merozoites opposite to the nucleus when antibodies against the INS-15 domain I were used in immunofluorescence microscopy. When the antibodies against the INS-15-specific polypeptide were used, the expression of INS-15 appeared to be more confined in both sporozoites and merozoites. Thus, INS-15 and INS-16 may be expressed in different organelles of the parasites.

INS-15 protein could be possibly involved in peptide degradation in *C. parvum*. As predicted *in silico*, INS-15 has four conserved domains common to M16A proteases. The domain I contains an active site characterized by the zinc-binding motif HYLEH, while domains II and IV have homology to the M16A inactive catalytic domains of insulin-like proteases, suggests that INS-15 is a functional M16A protease [24]. *Pf*SPP, an M16C protein in *P. falciparum*, cleaves the transit peptide of plastid-targeted proteins [21,25,26]. Falcilysin is another M16C protein in *P. falciparum* and was shown to degrade transit peptide in the apicoplast [20]. Although *Cryptosporidium* spp. have lost the apicoplast, INS-15 might also participate in peptide degradation in *C. parvum*. In addition, the INS-15 protein is expressed from sporozoites to 48 h *in vitro* culture. This suggests that INS-15 may exert its functions in multiple life cycle stages, including the initial invasion of host cells by sporozoites, parasite multiplication after the entry, and secondary invasion of neighboring cells by merozoites.

There appears to be proteolytic processing of INS-15 during its expression in *E. coli*. Multiple products were observed in the SDS-PAGE analysis of the recombinant protein expressed in *E. coli*. Attempts were made to improve the expression of the full-length INS-15 using BL21-Codon plus(DE3)-RIPL cells or optimizing the codons of *cgd3_4260* to those of *E. coli*. However, no significant improvements in protein expression were observed. This was also observed in the expression of human insulinase in *E. coli* [27]. In western blot analysis of the native INS-15 protein in *C. parvum* sporozoites, several lower bands with molecular weight of ~60 kDa, ~38 kDa, and ~23 kDa were also observed. It is, therefore, possible that the INS-15 sequence contains proteolytic cleave sites. A pro-domain cleave site, SΦXE/D (in which Φ is hydrophobic and X is any amino acid), which was first characterized in ROP1 [28,29], is present in toxolysin-1 [15]. INS-15 apparently has two such cleavage sites, with the sequence SIID in amino acids 375–378 and SIRD in amino acids 923–926. In theory, their cleavage in the full-length INS-15 protein could generate fragments of ~29 kDa, 40 kDa and 64 kDa, which are similar in size to INS-15 protein fragments we observed. Taken together, the INS-15 protein may be post-translationally processed to exert its multiple biological activities in ways similar to toxolysins.

## 4. Materials and Methods

### 4.1. Parasite and Cell Line

Oocysts of *C. parvum* IOWA isolate were purchased from Waterborne, Inc. (New Orleans, LA, USA) and stored at 4 °C for less than two months since their harvest. Prior to use, they were treated with 0.5% sodium hypochlorite on ice for 10 min and washed three times with phosphate-buffered saline (PBS) by centrifugation. Genomic DNA was extracted from *C. parvum* oocysts using the Qiagen DNeasy Blood & Tissue Kit (Qiagen, Hilden, Germany).

Human ileocecal adenocarcinoma HCT-8 cells were obtained from the Chinese Academy of Sciences Shanghai Branch. For *in vitro* experiments, HCT-8 cells were seeded into 12-well cell culture plates and cultured in RPMI 1640 medium containing 10% fetal bovine serum (FBS), 100 units/mL penicillin and 100 µg/mL streptomycin at 37 °C until ~90% confluence. Hypochlorite-treated oocysts were suspended in 2% FBS-supplemented RPMI 1640 medium and added into the plates at 5 × 10^5^ oocysts/well. After incubation for 2 h, uninvaded parasites were washed off the culture with PBS. Fresh 2% FBS-supplemented RPMI 1640 medium was added to the culture, which was maintained for a specified duration depending on the assay.

### 4.2. Cloning, Expression and Purification of INS-15 and INS-15 Domain I

Domains within INS-15 (*cgd3_4260*) were predicted using Pfam 31.0 (http://pfam.xfam.org). The full *cgd3_4260* gene was amplified from genomic DNA of *C. parvum* IOWA isolate using primers 5′-GGCCCATGGGGTGTATTTCATTATTA-3′ (the NcoI restriction site underlined) and 5′-GGCCTCGAGTATTGCATTAAAAACATTC-3′ (the XhoI restriction site underlined), while its domain I region was amplified using 5′-CGCGGATTCAGATATATTAAGTTGA-3′ (the BamHI restriction site underlined) and 5′-CCGCTCGAGGATTAAAACGAAAG-3′ (the XhoI restriction site underlined). The PCR was performed in a GeneAmp 9700 (Applied Biosystems) with the following cycling condition: 95 °C for 5 min, 35 cycles of 95 °C for 45 s, 55 °C for 45 s, and 72 °C for 105 s, and 72 °C for 7 min. The PCR products generated were cloned into the pET-28a vector (Novagen, Madison, WI, USA). The recombination plasmids generated were used to transform *Escherichia coli* DH5α cells. The bacterial colonies were screened by PCR using the T7/T7t universal primers, with positive colonies being sequenced to confirm their identity and sequence accuracy.

The plasmid containing the correct sequence of the *cgd3_4260* gene or the domain I fragment was transformed into *E. coli* BL21(DE3) cells or Rosetta(DE3) cells for protein expression. In some experiments, codons in the *cgd3_4260* gene were modified to *E. coli* ones using the gene synthesis approach prior to the transformation of the BL21-Codon plus(DE3)-RIPL cells. The BL21(DE3) cells were cultured in LB medium containing 100 µg/mL kanamycin at 37 °C, while the Rosetta(DE3) or BL21-Codon plus(DE3)-RIPL cells were cultured in LB medium containing 100 µg/mL kanamycin and 34 µg/mL chloramphenicol at 37 °C. After the OD_600_ reached 0.6–0.8, the expression of INS-15 or INS-15 domain I in *E. coli* was induced by adding 0.1 mM IPTG to the culture at 18 °C for 8 h. The expression level of the target protein was evaluated using the SDS-PAGE with Coomassie blue G-250 staining.

For the purification of INS-15 and INS-15 domain I, cultured *E. coli* cells were harvested by centrifugation and lysed by sonication on ice. The lysate was centrifuged and the pellet was dissolved in PBS buffer containing 8 M urea. After centrifugation again, the supernatant was filtered through a 0.45-µm cellulose acetate membrane filter (Millipore, Billerica, MA, US), and loaded onto Ni-NTA beads (Novagen, Madison, WI, USA) at 16 °C and 90 rpm for 4 h. The beads were washed with 10 volumes of 6 M PBS-buffered urea containing 20 mM imidazole and eluted with 6 volumes of 6 M PBS-buffered urea containing 250 mM imidazole. The purified protein was examined using SDS-PAGE with Coomassie blue G-250 staining and analyzed for identity using Matrix-Assisted Laser Desorption/Ionization Time of Flight Mass Spectrometry (MALDI-TOF-MS).

### 4.3. Preparation of Antibodies Against INS-15 Domain I and Polypeptide

Polyclonal antibodies against the INS-15 domain I and an INS-15 specific polypeptide (amino acid sequence CEITTKTYDFDWKKN selected based on sequence alignment of INS-15 and INS-16 (https://www.ebi.ac.uk/Tools/msa/clustalo/)) (Figure 1b) were raised in pathogen-free rabbits by Genscript Ltd. (Nanjing, China). Three hundred micrograms of the recombinant protein or polypeptide conjugated with keyhole limpet hemocyanin were emulsified with Freund’s complete adjuvant and used in the intramuscular immunization of rabbits. Two subsequent booster immunizations were administered at 2-week intervals with 300 µg of each antigen emulsified in Freund’s incomplete adjuvant. Two weeks after the last immunization, the rabbits were sacrificed, sera collected, and the polyclonal IgG antibodies purified using an affinity chromatographic column conjugated with INS-15 domain I or INS-15 polypeptide.

### 4.4. Western Blot Analysis of Native INS-15

To assess the expression of the native INS-15, oocysts treated with 0.5% sodium hypochlorite were suspended in PBS buffer containing 0.75% taurodeoxycholic acid and 0.25% trypsin and incubated at 37 °C for 1 h. The sporozoites released were collected by centrifugation and resuspended in PBS containing 1% protease inhibitor cocktail (Merck, Darmstadt, Germany). They were lysed by adding a protein-loading buffer (Yeasen, Shanghai, China) and boiling for 5 min. Proteins (from ~5 × 10^6^ sporozoites/lane) in the lysate were separated on SDS-PAGE and transferred onto nitrocellulose membranes. The latter were blocked with 5% nonfat milk-PBST for 2 h and incubated with anti-INS-15 domain I antibodies (~0.5 µg/mL), anti-INS-15 polypeptide antibodies (~1.5 µg/mL), or pre-immune serum (1:1000) for 2 h. Horseradish peroxidase (HRP)-conjugated goat-anti-rabbit antibodies (Earthox, Millbrae, CA, USA) were used at 1:5000 as the secondary antibodies in the western blot analysis. After 1-h incubation and three washes with PBST, the blots were treated with an enhanced chemiluminescent reagent (Thermo Fisher, Rockford, IL, USA) and analyzed with a Tanon 5200 (Tanon, Shanghai, China).

### 4.5. Quantitative Analysis of cgd3_4260 Gene Expression

The relative expression levels of the *cgd3_4260* gene in intracellular parasites developed in HCT-8 cells for 2–72 h were evaluated by qPCR. Parallel data on the expression of the 18s rRNA (Cp18s) gene of *C. parvum* were used in data normalization. Each of the qPCR reactions contained 0.1 mM primers, 1 µL of cDNA synthesized from 2 µg of total RNA using the GoScript Reverse Transcription System (Promega, Beijing, China), and 10 µL of SYBR Green PCR Mix (TOYOBO, Osaka, Japan) in a 20-µL reaction. The PCR reaction was conducted on a LightCycler 480 (Roche, Basel, Switzerland), with the following cycling conditions: 95 °C for 3 min and 45 cycles of 95 °C for 30 s, 58 °C for 30 s, and 72 °C for 30 s. The primers used included 5′-AGCCGCACTTTCGATGTTTT-3′ and 5′-ATCTGATAGCTCGTGAGTCAC-3′ for the *cgd3_4260* gene (amplicon: 154 bp), and 5′-CTCCACCAACTAAGAACGGCC-3′ and 5′-TAGAGATTGGAGGTTGTTCCT-3′ for the 18s rRNA gene (amplicon: 256 bp) [18]. The expression level of the *cgd3_4260* gene in infected HCT-8 cells was calculated using the 2^−△△CT^ method [30]. The data presented were from three independent experiments performed in duplicate.

### 4.6. Immunofluorescence Assay (IFA)

Sporozoites resuspended in PBS were dried onto microscope slides, whereas intracellular stages of *C. parvum* in HCT-8 cells were grown on coverslips for 24 and 48 h. These slides or coverslips were fixed with methanol at room temperature for 15 min. After three washes with PBS, the fixed sporozoites or cells were treated with 0.5% Triton-X in PBS for 15 min and blocked for nonspecific binding with 5% BSA in PBS for 1 h. After three washes with PBS, they were incubated with antibodies against INS-15 domain I (~0.6 µg/mL) or INS-15 polypeptide (~3.9 µg/mL) in 5% BSA-PBS for 1 h, followed by incubation with Alexa Fluor 594-conjugated Goat Anti-rabbit IgG (Cell Signaling Technology, Beverly, MA, US) in 5% BSA-PBS at 1:400 for another hour. After three washes, the slides or coverslips were counterstained with the nuclear stain 4′,6-diamidino-2-phenylindole (DAPI, Sigma, St Louis, MO, USA). After three more washes, the slides or coverslips were mounted with No-Fade Mounting Medium (Booster, Wuhan, China) and examined using differential interference contrast (DIC) and fluorescence microscopy on a BX53 microscope (Olympus, Tokyo, Japan).

### 4.7. Invasion Neutralization Assay

Hypochlorite-treated oocysts were incubated in culture medium containing increasing dilutions of polyclonal antibodies or pre-immune serum at 37 °C for 15 min, with a culture medium only as the negative control. The mixture was inoculated onto HCT-8 cells grown on coverslips to ~90% confluence in 12-well plates as described above. After 2-h incubation, the cells were washed three times and incubated for an additional 22 h in fresh culture medium. The coverslips were blocked with 5% BSA in PBS, stained with Cy3-labeled Sporo-Glo antibodies (Waterborne), and examined under the BX53 immunofluorescence microscope. Images of 50 microscope fields per coverslip were captured randomly under 200×, and the number of the parasites in the fields was quantified using ImageJ (https://imagej.nih.gov/ij/). The percent of inhibition of infection was calculated using the following formula: (1 − [No. of parasites after antibody treatment/No. of parasites after pre-immune serum treatment]) × 100%. The mean percent inhibition was calculated based data from three independent experiments. A paired *t*-test implemented in SPSS Statistics Version 23.0 (IBM Corp., Armonk, NY, USA) was used to compare the means of two groups.

## 5. Conclusions

We have expressed an INS of *C. parvum*, INS-15, one of the few INS in the parasite that have all four domains of classic INS. Our results indicated that INS-15 is expressed in the mid-anterior region of sporozoites and the area of merozoites opposite to the nucleus, and antibodies against its first domain partially neutralized the invasion of host cells. Further studies are needed to differentiate the neutralization effects of antibodies against INS-15 from those by antibodies against INS-16 before we have a better appreciation of the biological functions of INS in *Cryptosporidium* spp.
